# Assessment of Low Bone Mineral Density in Untreated Patients with Takayasu's Arteritis

**DOI:** 10.1155/2021/6489631

**Published:** 2021-10-13

**Authors:** Lingfei Mo, Jing Wang, BoMiao Ju, Yanhua Wang, Jing Luo, Juan Tian, Lan He

**Affiliations:** Department of Rheumatology, First Affiliated Hospital of Xi'an Jiaotong University, Xi'an, Shaanxi 710061, China

## Abstract

Chronic inflammation affects bone metabolism and accelerates bone loss. This study is aimed at analyzing the prevalence of low bone mineral density (LBMD) in patients with untreated Takayasu's arteritis (TA) and risk factors. Forty untreated TA patients were enrolled, including 38 premenopausal women and 2 men before 50 years old. The control group included 60 age- and gender-matched healthy persons. Bone mineral density (BMD) of lumbar vertebrae and hip in patients with TA and the control group was measured by the dual-energy X-ray method. Serum 25OHD and *β*-CTX were also measured. The lumbar BMD of TA patients (0.89 ± 0.11 g/cm^2^) was significantly lower than that of the healthy control (0.97 ± 0.11 g/cm^2^). The prevalence of LBMD at the lumbar spine (17.50%) was significantly higher than that of the control group (3.33%). However, there was no significant difference at the hip. The 25OHD of TA patients was lower than that of healthy controls, while the level of *β*-CTX was higher. The levels of total cholesterol (TC), low-density lipoprotein cholesterol (LDL-C), and high-density lipoprotein cholesterol (HDL-C) in patients with LBMD were higher than those in patients with normal BMD. According to univariate correlation analysis, there was a significant negative correlation between LDL-C and lumbar BMD. Binary logistic regression analysis showed that LDL-C was an important factor affecting the occurrence of LBMD in patients with TA (OR = 25.269, *P* = 0.02). Our result reveals bone loss in TA patients, which hints the relationship among inflammation, lipid metabolism, and bone metabolism.

## 1. Introduction

Osteoporosis is a systemic bone metabolic disease characterized by low bone mass, microarchitectural deterioration of bone tissue, decrease of bone strength, and high susceptibility to fracture [1]. Usually, osteoporosis is common in postmenopausal women and elderly men. Unfortunately, increasing studies have shown that young patients with inflammatory diseases are at high risk [2]. Up to now, no consensuses are reached on the diagnostic criteria for osteoporosis in premenopausal women [3]. According to the literature, *Z*-scores are recommended for premenopausal women. “Below the expected range for age” is defined when the *Z*-score is lower than -2.0 standard deviation (SD) [4]. Consistently, low bone mineral density (LBMD) is employed to represent “below the expected range for age.” Previous studies have suggested a high prevalence of low bone mineral density and osteoporosis in patients with systemic lupus erythematosus (SLE) [5]. The occurrence of LBMD and osteoporosis in patients with SLE was 25-75% and 10-68%, respectively [6]. A population-based study of 7332 SLE patients in the UK, with a 28079 age/sex-matched control group, showed that the incidence of osteoporosis in SLE increased by 2.53 times [7]. Similarly, the proportion of osteoporosis with rheumatoid arthritis was 13% for premenopausal female patients but went up to 50% for postmenopausal counterparts [8]. In our previous study, the incidence of LBMD and osteoporosis was 35.5% and 25.2% for patients with connective tissue diseases, respectively [9].

Inflammation is an important factor for causing bone loss. The pathogenesis of inflammation-induced bone loss mainly relates to proinflammatory cytokines, lymphocytes, osteoclasts, and osteoblasts. RANK-RANKL-OPG and Wnt/*β*-catenin are the two main pathways for inflammation-induced bone loss [10, 11]. Takayasu's arteritis (TA) is a chronic inflammatory disease, which mainly involves large blood vessels, especially the aorta and its main branches [12]. Inflammatory biomarkers such as CRP, ESR, and IL-6 are elevated in TA patients. Thus, it is highly speculated that patients with TA may also have a similar bone loss. Unfortunately, few reports focus on this aspect until now. Herein, this study is aimed at investigating the incidence of LBMD in untreated patients with TA and the corresponding risk factors.

## 2. Materials and Methods

### 2.1. Subjects

This study was approved by the Ethics Committee of the hospital. From January 1st 2013 to January 1st 2020, 40 untreated patients with TA were enrolled from the Department of Rheumatology of the First Affiliated Hospital of Xi'an Jiaotong University, which is the biggest general hospital under the direct administration of the Chinese Ministry of Health. Inclusion criteria include (i) less than 50 years old, (ii) male or premenopausal female patients, and (iii) fulfillment of the diagnosis of TA according to the 1990 American College of Rheumatology criteria [13]. Exclusion criteria include subjects (i) with concomitant chronic infections, bone fracture, endocrine system diseases, other autoimmune diseases, or major organ failure and (ii) taking glucocorticoids, immunosuppressants, anticoagulation drugs, antiosteoporosis drugs, and lipid regulating drugs six months before. In addition, 60 healthy subjects, matched sex, age, and body mass index (BMI), were selected as the control group.

### 2.2. Research Methods

The demographic data of the research subjects were recorded, including the patient's gender, age, course of disease, and body mass index (BMI). The results of laboratory tests were collected including blood calcium, phosphorus, serum alkaline phosphatase (ALP), 25-hydroxyvitamin D (25OHD), parathormone (PTH), *β*-cross-linked C-telopeptide of type 1 collagen (*β*-CTX), C-reactive protein (CRP), erythrocyte sedimentation rate (ESR), total cholesterol (TC), triglycerides (TG), high-density lipoprotein cholesterol (HDL-C), and low-density lipoprotein cholesterol (LDL-C). The evaluation of disease activity for TA was based on the Kerr score of NIH standards [14] and the Indian Takayasu Clinical Activity Score (ITAS2010) [15].

#### 2.2.1. BMD Measurements

BMD (bone mineral density) at the lumbar spine (anteroposterior, L1–L4) and total hip (left femoral neck) was tested using dual-energy X-ray absorptiometry (LEXXOS-LX381, French DMS). All measurements were performed according to standard instrument procedures. Here, all BMD results were expressed in absolute values (g/cm^2^) and *Z*-score for L1–L4 and total hip.

### 2.3. Statistical Analysis

SPSS 23.0 was employed in statistical data analysis. Continuous variables with normal distribution were represented as the mean ± SD, while nonnormal ones were reported as median (interquartile range). The Shapiro-Wilk test was used to evaluate normality and homogeneity of variance. Parametric and nonparametric variables were analyzed by the Student *t*-test and Mann-Whitney *U-*test. For comparison of the frequencies of categorical variables, Fisher's exact test was performed. The associations between variables were examined by Pearson's correlation analysis or Spearman's correlation analysis. The significance level was set at *P* < 0.05.

## 3. Results

### 3.1. Clinical Characteristics of Patients with TA and Healthy Control

The clinical data are shown in Table 1, where 40 newly diagnosed untreated TA patients are enrolled. All of them are nonsmokers. The median score of the activity for TA was determined to be 3, according to the KERR score. When ITAS2010 was used to assess disease activity, the average score was 12.18. This means that most of them had active disease at the time of diagnosis.

### 3.2. Bone Turnover Markers in TA

The bone turnover markers in TA were assessed by comparing the untreated patients with the healthy control. As showed in Table 2, 25OHD levels decreased significantly in TA patients when compared with the control group. On the contrary, *β*-CTX inclined remarkably in the TA patients.

### 3.3. BMD and Incidence of LBMD in TA

The outcomes of BMD measurements are listed in Table 2. For TA patients, the mean BMD was determined to be 0.89 ± 0.11 g/cm^2^ at the lumbar spine, significantly lower than that of the healthy control. However, no big differences were found with respect to mean BMD (0.93 (0.87~0.99) g/cm^2^) between TA patients and healthy control at the total hip. Then, the incidence of LBMD was 17.5%, much higher than that of the healthy control (*P* = 0.028).

### 3.4. Characteristics of Patients with LBMD

To investigate the causes of bone loss in TA, TA patients with normal BMD or with LBMD at the lumbar spine or total hip are compared (Table 3). Obviously high levels of TC, LDL-C, and HDL-C were found in the LBMD group in comparison with the normal BMD group.

### 3.5. Association of Clinical Parameters and LBMD

**Figure 1:**
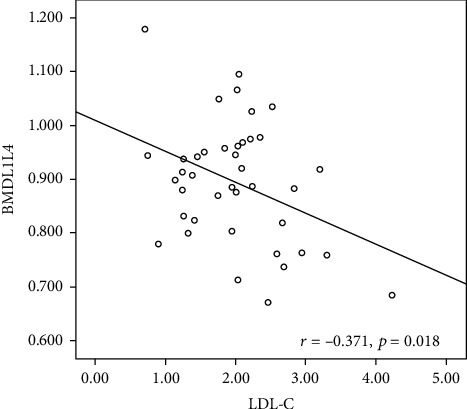
The negative correlation between LDL-C and BMD at the lumbar spine in TA patients. TA = Takayasu's arteritis; BMD = bone mineral density; LDL-C = low-density lipoprotein cholesterol.

Among 40 TA patients at the time of diagnosis, a negative correlation was obtained between LDL-C and BMD at the lumbar spine (Figure 1), according to univariate correlation analyses. Then, TC, LDL-C, and HDL-C were associated with LBMD in TA patients based on binary logistic regression analysis (Table 4). However, 25OHD fails to correlate with BMD, ESR, CRP, ITAS2010, and KERR nor *β*-CTX.

## 4. Discussion

Several studies have shown that bone loss is related to autoimmune diseases, such as SLE, RA, inflammatory myositis, and other connective diseases [10, 16, 17]. Until now, however, few studies focus on bone loss in TA. According to the literature [18], TA is first diagnosed among Japanese women and attacks Asians frequently, especially in women below the age of 40. According to our knowledge, this is the first report showing a high occurrence of LBMD in untreated TA patients, which can be associated with abnormal serum lipid spectrum.

As menopausal status or age greatly affects bone metabolism, our study enrolled premenopausal female patients and male patients younger than 50. They are also susceptible to TA. According to the guideline [19], BMD “below expected range for age” is defined by a *Z*‐score ≤ −2.0 SD for premenopausal females and males < 50 years.

Cramarossa et al. [20] used *Z*-score to evaluate bone loss in inception patients of SLE. In their cohort, 17.3% premenopausal females (*n* = 173; mean age 31 ± 8 years) were cataloged to be “below the expected range for age,” similar to 17.5% in our study. Souto et al. [21] also used *Z*-score to evaluate bone loss in SLE patients, and this percentage was found to be 25% (21/84). Here, its high value may be due to the application of glucocorticoids and long disease duration.

Vitamin D plays an important role in bone metabolism and inflammation. In this work, it is observed that serum 25OHD levels are significantly lower in TA patients than those in healthy control. Alibaz-Oner et al. [22] found that serum 25OHD levels were significantly lower in TA patients (16.93 ± 10.62 nmol/L) when compared with the healthy control (64.63 ± 21.82 nmol/l, *P* = 0.001). They did not provide any association between vitamin D levels and acute-phase reactants. Liao et al. [23] also observed high prevalence of vitamin D deficiency in TA patients (11.8 ± 4.7 *μ*g/L versus 23.2 ± 8.3 *μ*g/L in healthy control, *P* < 0.001). In their study, serum 25OHD levels were negatively correlated with IL-6 (*r* = −0.296, *P* = 0.023). According to our present outcomes, however, there are no hints to correlate 25OHD levels with BMD, ESR, CRP, ITAS2010, and KERR.

The terminal peptide of type I collagen is a nonhelical region peptide chain cross-linked at the end of type I collagen. As a specific product of osteoclast degradation during bone resorption, it exists in the form of amino-terminal peptide (NTX)/carboxyl terminal peptide (CTX) in the blood, reflecting the activity of osteoclasts. We observe that serum *β*-CTX in TA patients is significantly higher than that in healthy control, although there is no report on this relationship. Previous studies showed no relationship between *β*-CTX and disease activity of SLE [24, 25]. Consistently, it is unable to correlate BMD, ESR, CRP, ITAS2010, or KERR with serum *β*-CTX in our study.

Patients with LBMD exhibit higher levels of serum TC, LDL-C, and HDL-C than the normal BMD group, based on the present observation. According to univariate correlation analysis, LDL-C has negative correlations with BMD at the lumbar spine. Based on binary logistic regression analysis, TC, LDL-C, and HDL-C are associated with LBMD in TA patients. In recent years, previous studies have demonstrated a positive relationship between bone loss and abnormal lipid metabolism. Pelton et al. [26] found that the increase of cholesterol was evidently correlated with the decrease of bone mass in the mouse model. Adami et al. [27] found that lumbar spine and hip BMD *Z*-score values were negatively correlated with HDL cholesterol and Apo A lipoprotein, but positively related to LDL cholesterol, Apo B lipoprotein, and triglycerides. Most of these correlations remained statistically significant when BMD values were adjusted for body weight and BMI. Kim et al. [28] found that BMD values in lumbar vertebrae, pelvis, and femoral neck were inversely correlated with atherogenic dyslipidemia in South Korean men.

Up to now, the reason for the decrease of serum 25OHD and BMD in TA is unclear. Inflammation may be a key point, where a fine balance exists between disease activity and bone metabolism. The levels of IL-1 *β*, IL-6, and tumor necrosis factor (TNF) in TA increase significantly. By upregulating the receptor activator of nuclear factor kappa-B ligand (RANKL), these inflammatory factors promote the differentiation and maturation of osteoclast, as well as its increased activity. Consequently, this enhances bone resorption [11].

Osteoblasts and adipocytes are derived from bone marrow mesenchymal stem cells. Two pathways regulate the transformation between these two kinds of cells. One is the Wnt/*β*-catenin signal pathway, while the other one is the PPAR *γ* pathway. There is a crosstalk between them [29]. With respect to Wnt pathways, both classical and nonclassical ones can promote osteogenesis and inhibit the differentiation of bone marrow mesenchymal cells into adipocytes [30]. In contrast, the differentiation of bone marrow mesenchymal stem cells into adipocytes is promoted by the PPAR *γ* pathway, while differentiation towards osteoblasts is inhibited [31]. There is an inverse correlation between adipogenesis and osteoblastogenesis in bone marrow stromal cells, and a relation between serum lipids and bone mass is highly expected.

The major limitation of our study is that it is a cross-sectional study, so we cannot figure out the causal relationship among inflammation, serum lipids, and bone loss. To avoid the effects of age, menstruation, and glucocorticoids on BMD and blood lipids, our patients are treatment-naive premenopausal women and men below 50 years old. As consequence, the sample size is a bit small. A large sample and prospective study would be helpful to further clarify the relationship among inflammation, lipid metabolism, and bone metabolism.

In conclusion, our findings suggest that TA patients have a higher risk of LBMD than healthy control, together with lower levels of serum 25OHD and higher levels of *β*-CTX. The univariate correlation analyses revealed negative correlations between LDL-C and BMD at the lumbar spine. Binary logistic regression analysis shows that TC, LDL-C, and HDL-C are associated with LBMD in TA patients. Our result reveals bone loss in TA patients, which hints the relationship among inflammation, lipid metabolism, and bone metabolism.

## Figures and Tables

**Table 1:** Clinical data of the TA patients and the control.

Variable	TA (*n* = 40)	Healthy control (*n* = 60)	*P* value
Age (years)	30.48 ± 6.87	32.90 ± 6.45	0.076
Female (%)	38 (95%)	57 (95%)	1
BMI (kg/m^2^)	20.55 ± 2.09	21.10 ± 2.06	0.197
Disease duration (months)	6.00 (1.00~21.00)	—	
ESR (mm/h)	33.50 (16.25~66.50)	—	
CRP (mg/L)	10.70 (0~31.95)	—	
KERR score	3 (2~3)	—	
ITAS2010	12.18 ± 5.89		

TA = Takayasu's arteritis; BMI = body mass index; ESR = erythrocyte sedimentation rate; CRP = C-reactive protein; ITAS2010 = Indian Takayasu Clinical Activity Score.

**Table 2:** BMD and bone turnover markers in TA patients and healthy controls.

Variable	TA (*n* = 40)	Healthy controls (*n* = 60)	*P* value
25OHD^#^	10.30 (7.83~13.69)	16.00 (11.75~21.93)	0.000
*β*-CTX^#^	527.50 (372.00~617.48)	380.63 (257.10~468.08)	0.001
BMD L1-L4 (g/cm^2^)^∗^	0.89 ± 0.11	0.97 ± 0.11	0.002
BMD total hip (g/cm^2^)^#^	0.93 (0.87~0.99)	0.93 (0.87~1.04)	0.456
Number of LBMD^$^	7 (17.50%)	2 (3.33%)	0.028

^∗^The Student *t*-test; ^#^Mann-Whitney *U*-test; ^$^Fisher's exact test. TA = Takayasu's arteritis; BMD = bone mineral density; 25OHD = 25-hydroxyvitamin D; *β*-CTX = *β*-cross-linked C-telopeptide of type 1 collagen; LBMD = low bone mineral density.

**Table 3:** Characteristics of TA patients with normal BMD or with LBMD at the lumbar spine or total hip.

Variable	Normal BMD (*n* = 33)	LBMD (*n* = 7)	*P* value
Age (years)^∗^	31.09 ± 7.11	27.57 ± 5.03	0.223
BMI (g/cm^2^)^∗^	20.65 ± 2.22	20.09 ± 1.39	0.522
Disease duration (months)^#^	6.00 (1.00~18.00)	1.00 (1.00~36.00)	0.419
ESR (mm/h)^#^	27.00 (16.50~67.00)	51.00 (7.00~65.00)	0.929
CRP (mg/L)^#^	10.10 (0~32.20)	16.40 (0~32.10)	0.730
KERR^#^	3 (2~3)	3 (2~3)	0.775
ITAS2010^∗^	11.70 ± 5.97	14.43 ± 5.32	0.270
25OHD^#^	9.20 (7.25~12.31)	14.02 (10.33~17.79)	0.057
*β*-CTX^#^	513.30 (372.60~597.20)	563.10 (289.20~1049.00)	0.403
TC (mmol/L)^#^	2.68 (3.21~3.64)	4.21 (3.85~5.00)	0.001
LDL-C (mmol/L)^∗^	1.80 ± 0.59	2.89 ± 0.71	0.000
HDL-C (mmol/L)^∗^	0.96 ± 0.26	1.28 ± 0.36	0.008
TG (mmol/L)^#^	0.79 (0.61~1.14)	0.91 (0.80~1.71)	0.176

CRP = C-reactive protein; ESR = erythrocyte sedimentation rate; TC = total cholesterol; LDL-C = low-density lipoprotein cholesterol; HDL-C = high-density lipoprotein cholesterol; TG = triglyceride; ITAS2010 = Indian Takayasu Clinical Activity Score. ^∗^The Student *t*-test; ^#^Mann-Whitney *U*-test.

**Table 4:** Clinical correlations of BMD at the lumbar spine by univariate and multivariate analyses for TA patients.

Clinical characteristic	Univariate HR (95% CI)	*P* value	Multivariate HR (95% CI)	*P* value
TC (mmol/L)	11.348 (1.796~71.698)	0.010	0.747 (0.046~12.194)	0.838
LDL-C (mmol/L)	21.448 (2.155~213.488)	0.009	25.269 (1.674~381.530)	0.020
HDL-C (mmol/L)	55.645 (1.767~1752.359)	0.022	64.651 (0.455~9180.025)	0.099

TA = Takayasu's arteritis; TC = total cholesterol; LDL-C = low-density lipoprotein cholesterol; HDL-C = high-density lipoprotein cholesterol.

## Data Availability

The data in the current study are available from the corresponding author on reasonable request.

## References

[1] Kanis J. A., Cooper C., Rizzoli R., Reginster J. Y. (2019). European guidance for the diagnosis and management of osteoporosis in postmenopausal women. *Osteoporosis International*.

[2] Briot K., Geusens P., Em Bultink I., Lems W. F., Roux C. (2017). Inflammatory diseases and bone fragility. *Osteoporosis International*.

[3] Rozenberg S., Bruyère O., Bergmann P. (2020). How to manage osteoporosis before the age of 50. *Maturitas*.

[4] Camacho P. M., Petak S. M., Binkley N. (2020). American Association of Clinical Endocrinologists/American College of Endocrinology Clinical Practice Guidelines for the Diagnosis and Treatment of Postmenopausal Osteoporosis--2020 Update. *Endocrine Practice*.

[5] Salman-Monte T. C., Torrente-Segarra V., Vega-Vidal A. L. (2017). Bone mineral density and vitamin D status in systemic lupus erythematosus (SLE): a systematic review. *Autoimmunity Reviews*.

[6] Edens C., Robinson A. B. (2015). Systemic lupus erythematosus, bone health, and osteoporosis. *Current Opinion in Endocrinology, Diabetes, and Obesity*.

[7] Rees F., Doherty M., Grainge M., Lanyon P., Davenport G., Zhang W. (2016). Burden of comorbidity in systemic lupus erythematosus in the UK, 1999-2012. *Arthritis Care & Research (Hoboken)*.

[8] Adami G., Saag K. G. (2019). Osteoporosis pathophysiology, epidemiology, and screening in rheumatoid arthritis. *Current Rheumatology Reports*.

[9] Feng X., Mo L., Ju B. (2016). Prevalence and possible risk factors of low bone mineral density in untreated patients with connective tissue diseases. *Chinese Journal of Osteoporosis*.

[10] Bultink I. E., Vis M., van der Horst-Bruinsma I. E., Lems W. F. (2012). Inflammatory rheumatic disorders and bone. *Current Rheumatology Reports*.

[11] Bultink I. E. M. (2018). Bone disease in connective tissue disease/systemic lupus erythematosus. *Calcified Tissue International*.

[12] Numano F., Okawara M., Inomata H., Kobayashi Y. (2000). Takayasu's arteritis. *The Lancet*.

[13] Arend W. P., Michel B. A., Bloch D. A. (1990). The American College of Rheumatology 1990 criteria for the classification of Takayasu arteritis. *Arthritis and Rheumatism*.

[14] Kerr G. S., Hallahan C. W., Giordano J. (1994). Takayasu arteritis. *Annals of Internal Medicine*.

[15] Misra R., Danda D., Rajappa S. M. (2013). Development and initial validation of the Indian Takayasu Clinical Activity Score (ITAS2010). *Rheumatology*.

[16] So H., Yip M. L., Wong A. K. (2016). Prevalence and associated factors of reduced bone mineral density in patients with idiopathic inflammatory myopathies. *International Journal of Rheumatic Diseases*.

[17] Adami G., Fassio A., Rossini M. (2019). Osteoporosis in rheumatic diseases. *International Journal of Molecular Sciences*.

[18] Seyahi E. (2017). Takayasu arteritis: an update. *Current Opinion in Rheumatology*.

[19] Camacho P. M., Petak S. M., Binkley N. (2016). American Association of Clinical Endocrinologists and American College of Endocrinology Clinical Practice Guidelines for the Diagnosis and Treatment of Postmenopausal Osteoporosis -- 2016. *Endocrine Practice*.

[20] Cramarossa G., Urowitz M. B., Su J., Gladman D., Touma Z. (2017). Prevalence and associated factors of low bone mass in adults with systemic lupus erythematosus. *Lupus*.

[21] Souto M. I., Coelho A., Guo C. (2012). The prevalence of low bone mineral density in Brazilian patients with systemic lupus erythematosus and its relationship with the disease damage index and other associated factors. *Journal of Clinical Densitometry*.

[22] Alibaz-Oner F., Asmaz-Haliloglu Ö., Gogas-Yavuz D., Can M., Haklar G., Direskeneli H. (2016). Vitamin D levels in Takayasu's arteritis and a review of the literature on vasculitides. *Journal of Clinical Laboratory Analysis*.

[23] Liao H., Pan L. L., du J., Wang T. (2018). Relationships between the levels of serum 25-hydroxyvitamin D and interleukin-6 in patients with Takayasu's arteritis. *Zhonghua Yi Xue Za Zhi*.

[24] Redlich K., Ziegler S., Kiener H. P. (2000). Bone mineral density and biochemical parameters of bone metabolism in female patients with systemic lupus erythematosus. *Annals of the Rheumatic Diseases*.

[25] Sarkissian A., Sivaraman V., Bout-Tabaku S. (2019). Bone turnover markers in relation to vitamin D status and disease activity in adults with systemic lupus erythematosus. *Lupus*.

[26] Pelton K., Krieder J., Joiner D., Freeman M. R., Goldstein S. A., Solomon K. R. (2012). Hypercholesterolemia promotes an osteoporotic phenotype. *The American Journal of Pathology*.

[27] Adami S., Braga V., Zamboni M. (2004). Relationship between lipids and bone mass in 2 cohorts of healthy women and men. *Calcified Tissue International*.

[28] Kim Y. H., Nam G. E., Cho K. H. (2013). Low bone mineral density is associated with dyslipidemia in South Korean men: the 2008-2010 Korean National Health and Nutrition Examination Survey. *Endocrine Journal*.

[29] Takada I., Kouzmenko A. P., Kato S. (2009). Wnt and PPAR*γ* signaling in osteoblastogenesis and adipogenesis. *Nature Reviews Rheumatology*.

[30] Ross S. E., Hemati N., Longo K. A. (2000). Inhibition of adipogenesis by Wnt signaling. *Science*.

[31] Kim J., Lee Y. J., Kim J. M. (2016). PPAR*γ* agonists induce adipocyte differentiation by modulating the expression of Lipin-1, which acts as a PPAR*γ* phosphatase. *The International Journal of Biochemistry & Cell Biology*.

